# Musculoskeletal disorders in eSports athletes: A systematic review

**DOI:** 10.1002/jeo2.70786

**Published:** 2026-05-25

**Authors:** Ludwig Schlesiger, Jakub Oronowicz, Christoph Lutter, Thomas Tischer, Romain Seil, Andrzej Jasina

**Affiliations:** ^1^ Clinic for Orthopaedics and Trauma Surgery Diako Hospital Bremen Bremen Germany; ^2^ Clinic for Orthopaedics and Trauma Surgery Malteser St. Mary's Hospital Erlangen Germany; ^3^ Clinic and Polyclinic for Orthopaedics Rostock University Medical Center Rostock Germany; ^4^ Luxembourg Institute of Research in Orthopaedics Sports Medicine and Science (LIROMS) Luxembourg City Luxembourg; ^5^ Department of Orthopaedic Surgery Centre Hospitalier de Luxembourg – Clinique d'Eich Luxembourg City Luxembourg

**Keywords:** competitive gaming, eSports, injury prevention, innovative sports, sports orthopaedics

## Abstract

**Purpose:**

With the growing popularity and professionalisation of eSports, concerns regarding the physical condition of professional e‐athletes are becoming the focus of attention. The potential effects of prolonged sitting and repetitive movements on the musculoskeletal system have not been fully researched. The aim of this review is to summarise the current scientific evidence on the prevalence of musculoskeletal disorders in professional eSports players and possible preventative measures.

**Methods:**

A systematic literature search was conducted following preferred reporting items for systematic reviews and meta‐analyses guidelines. All studies published from 2005 onwards investigating musculoskeletal disorders in professional e‐athletes were included. The quality of the studies was assessed using the Newcastle‐Ottawa Scale (NOS) adapted for cross‐sectional studies and case series.

**Results:**

Ten studies with a total of 789 e‐athletes met the inclusion criteria. Musculoskeletal disorders were highly prevalent, ranging from 26.8% to 86.2%. Commonly affected body regions included the lower back (4.7%–63.8%), shoulders (2.7%–59%), neck (11.3%–50%) and wrists (8%–44.8%). Eye fatigue was also reported (32.5%–92%). A significant association was found between longer training durations, inadequate ergonomic setup (e.g., absence of armrests) and the occurrence of musculoskeletal symptoms. Only a small proportion of e‐athletes sought medical consultation. The methodological quality of the included studies was moderate, with the majority employing a cross‐sectional design.

**Conclusions:**

Musculoskeletal disorders, frequently observed among e‐athletes, substantially impair performance. Current data emphasises the importance of preventive measures: ergonomic gaming stations, physical training and regular medical check‐ups. Further high‐quality prospective studies with large sample sizes and control groups are necessary to identify causal relationships and address them in clinical setup.

**Level of Evidence:**

Level IV, systematic review of predominantly cross‐sectional studies.

**Abbreviations**
BCTQ‐SSS: Boston Carpal Tunnel Questionnaire Symptom Severity Scale
BMI: body mass index
CI: confidence interval
EDI: equity, diversity and inclusion
HQ: high quality
IOC: International Olympic Committee
IPAQ: international physical activity questionnaire
LQ: low quality
MET: metabolic equivalent of task
MQ: moderate quality
NOS: Newcastle‐Ottawa Scale
NPRS: numeric pain rating scale
OR: odds ratio
PRISMA: preferred reporting items for systematic reviews and meta‐analyses
PROSPERO: international prospective register of systematic reviews

## INTRODUCTION

The global impact of video gaming is evidenced by the 3.2 billion gamers worldwide and the annual turnover of 347 billion US dollars [30, 31], with a continued rapid growth and expansion. In recent years, there has been a notable shift towards professionalisation of video gaming, accompanied by the advent of electronic sports, otherwise known as eSports [32]. This includes structured, competitive events in which professional eSports competitors, designated as e‐athletes, engage in individual or team‐based virtual competition on electronic devices, including game consoles or computers [11]. These events are conducted at a professional level and offer very high financial rewards. In 2013, 70 million viewers watched eSports on a global scale [11]. In 2019, this number increased to over 450 million, with a strong upward trend forecast [21]. The revenue generated by the eSport industry is in excess of 1 billion US dollars [9], with prize money amounting to 211 million US dollars in 2019, which illustrates the growing economic influence of eSports [5].

Despite the recognition of eSports as a sport by the International Olympic Committee (IOC) in 2017 [38], the official inclusion of eSports in the 2022 Asian Games [36] and the first Olympic Games for eSports to be held in Saudi Arabia by the IOC in 2027 [13], the question of whether eSports should be recognised as a sport remains controversial [3, 4, 5, 6, 7, 8, 9, 10, 11, 12, 13, 14, 15, 16, 17, 18, 19, 20, 21, 22, 23, 24, 25, 26, 27, 28, 29].

With daily training sessions of more than 5 h, of which approximately 1 h has been reported to be physical or fitness training, the total training volume of eSports athletes is comparable to traditional sports [2, 3, 4, 5, 6, 7, 8, 9, 10, 11, 12, 13, 14], but the effects on the musculoskeletal system are much less investigated. Few studies reported that intense practice of videogaming is associated with particularly negative musculoskeletal effects [15], with neck, shoulder and lower back pain [33] caused by the long‐lasting seated position in front of computers, mobile phones or screens. However, to date, scientific research remains limited regarding the presence of musculoskeletal effects in professional eSports players, although growing evidence of such effects among recreational video game users.

The aim of this systematic review is to provide a comprehensive summary and assessment of the existing scientific literature on the relationship between the prevalence of musculoskeletal disorders in professional eSports athletes, to facilitate a deeper comprehension of the medical challenges associated with athlete care. It was hypothesised that the prevalence of injuries in professional eSports athletes is high and that there is a need for specific prevention programs in the future.

## METHODS

This systematic review was registered in the PROSPERO database and conducted according to the Preferred Reporting Items for Systematic Reviews and Meta‐Analyses (PRISMA 2020) checklist [26]. The authors affirm their commitment to equity, diversity and inclusion in research. Efforts were made to ensure a comprehensive and unbiased literature search and data interpretation. The authorship team reflects a range of professional backgrounds and includes contributors from multiple countries. No barriers related to gender, ethnicity or other personal characteristics were applied in the conduct of this review.

### Search strategy

A systematic electronic search of English and German literature was performed on 31th July 2024 and updated on 16th March 2025 and 28th August 2025 to identify clinical studies that examined the association between participation in professional eSports and musculoskeletal disorders using PubMed and Embase databases. Additionally, the reference lists of included publications were manually searched to identify any further relevant articles.

The search strategy consisted two thematic blocks, the first of which covered eSports and the second of which covered musculoskeletal disorders. Individual terms were linked with Boolean operators (AND, OR). The complete search strategy for both databases was selected according to the following procedure: ‘(professional video gaming OR competitive video gaming OR esports OR esport OR e‐sports OR e‐sport) AND (musculoskeletal OR musculo‐skeletal OR musculosceletal OR musculo‐sceletal OR pain OR injury OR injuries)’.

### Inclusion criteria

In order to be included in this systematic review, publications had to fulfil the following criteria: The objective of the studies is to investigate the potential association between professional e‐athletes, participation in this activity and the occurrence of musculoskeletal disorders. Given the lack of a universally accepted definition of ‘professional’ in esports, we included studies in which participants were explicitly described as professional, elite, collegiate or nationally competitive players. Structured training with participation in official tournaments was required as an additional inclusion criterion. The resulting heterogeneity in competitive level is acknowledged and discussed in the Limitations section. The following publications were excluded from the review: those that did not concern the target population, focused exclusively on noncompetitive gaming, were published in a language other than German or English, were only available as abstracts or were considered grey literature or nonacademic studies. In addition, studies published before 2005 were excluded, as the professionalisation of eSports with the resulting development of professional tournaments and competitions occurred in the years that followed, and healthcare systems, medical knowledge and treatment guidelines have changed considerably over time. Consequently, studies published before 2005 may no longer be representative of the present day.

### Study selection, extraction and data synthesis

In order to guarantee a systematic and blinded screening procedure, the screening process was conducted using the Rayyan platform [25]. Once duplicates had been removed, the titles and abstracts of all articles were subjected to a screening process performed independently by two authors to determine their relevance in accordance with the established inclusion and exclusion criteria. In the event that no abstract was available, the full text of the article was obtained to assess its suitability for inclusion in the systematic review. The full text was then reviewed to determine its eligibility for inclusion in the review. All articles that were not excluded during the initial screening process underwent this process. All points of disagreement were discussed until a consensus was reached.

The specifics of each study were obtained utilising a predefined template in Microsoft Excel. The Excel template included article characteristics including authors, title, year, journal and region, as well as study characteristics including the study design, the topic studied and the database. Only the results from the integrated studies of relevance to musculoskeletal disorders were considered.

Following extraction, the data were synthesised narratively. Due to substantial heterogeneity regarding outcome definitions, recall periods, assessment tools and study populations, a quantitative meta‐analysis was deemed inappropriate. Therefore, a narrative synthesis was performed.

### Quality and risk of bias assessment

The Newcastle‐Ottawa Scales (NOS) adapted for cross‐sectional studies and adapted for case series were employed to evaluate the methodological quality of the included research and to assess the risk of bias for each study included in the systematic review [12, 14, 15, 16, 17, 18, 19, 20, 21, 22, 23]. This enabled the classification of scientific studies as either high‐ or low‐quality based on predefined cut‐off points. The NOS adapted for cross‐sectional studies comprises eight categories pertaining to methodological quality. While up to two points are awarded for two categories, a maximum of 10 points can be accumulated for each study. A score of 0–4 points was assigned to studies classified as unsatisfactory quality, 5–6 points to those of satisfactory quality, 7–8 points to those of good quality and 9–10 points to those of very good quality [12].

The NOS adapted for case series analyses nine categories in terms of methodological quality, with a maximum achievable score of nine points. A score of 0–3 points corresponds to studies classified as of low quality, 4–6 points to those of moderate quality (MQ) and 7–9 points to those of high quality [23].

The quality and risk of bias assessment of the included studies were independently assessed by two authors.

## RESULTS

**Figure 1:**
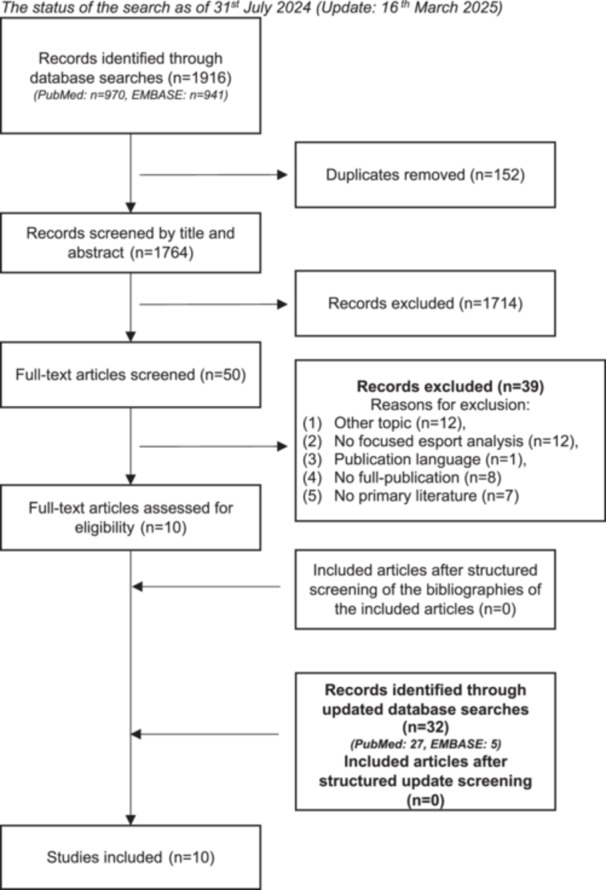
PRISMA flow chart of screening process. The status of the search as of 31st July 2024 (update: 16th March 2025). PRISMA, preferred reporting items for systematic reviews and meta‐analyses.

A systematic search of the literature was conducted both electronic and manual to identify articles that met the inclusion and exclusion criteria. The search found a total of 1943 articles, of which 10 met the inclusion criteria and were therefore included in this systematic review. The results of the search are presented in Figure 1.

A total of 789 individuals participated in all 10 studies, with the number of participants per study ranging from 39 to 188. Of the total number of participants, 630 were male and 54 were female, while gender was not specified in two studies. The age of the participants ranged from a mean of 17.1 ± 2.3 (range 15–35) to 24.6 ± 4.2 (range 18–40), with one study providing the age as a range and two studies categorising the participants into different age groups.

The 10 included studies consisted nine surveys, eight of which investigated the general prevalence of musculoskeletal disorders in professional eSports players [4, 5, 6, 7, 8, 9, 10, 11, 12, 13, 14, 15, 16, 17, 18, 19]. Another survey investigated the prevalence of carpal tunnel in professional eSports players [1]. Furthermore, a retrospective case series on posture, mobility and stability of the spine in professional eSports players was integrated into the study [17] (see Table 1).

**Table 1:** Included publications.

Author	Publication year	Region	Population	Sex	Age	Outcomes (primary/secondary)	Results (significant/not significant)	Newcastle‐Ottawa Scales
Clements et al.	2022	USA	153 collegiate eSports athletes	135 males, 18 females	mean: 21.0 ± 3.6	Injury prevalence in college athletes in relation to time spent training and competing	41 (26.8%) at least one injury; 17 (11.1%) multiple injuries. Longer time in team correlated with higher injury incidence (*p* = 0.03). More practice hours linked to higher injury incidence; 31.7% of injured athletes versus 10.7% of uninjured athletes practiced >5 h/day (*p* = 0.01). No significant differences in age, sex, scholarship status, age of starting eSports or weekly playing days. Injury distribution: 25 wrist (33.3%), 11 neck (14.7%), 10 back (13.3%), 9 finger (12%), 8 hand (10.7%), 6 elbow (8%), 4 thumb (5.3%), 2 shoulder (2.7%). 7 athletes missed 2–8 weeks of competition due to injury.	6
Lam et al.	2022	China	50 elite mobile‐eSports athletes	All males	Mean: 20.0 ± 1.67	Health risks and complaints	7 (14%) reported lumbar muscle strain. Pain by body region: neck (20, 40%, all mild), fingers (19, 38%, 15 mild, 4 moderate), head (16, 32%, 13 mild, 3 moderate), upper back (12, 24%, all mild), lower back (10, 20%, 8 mild, 2 moderate), shoulder (8, 16%, 7 mild, 1 moderate), wrist (4, 8%, all mild), knee (3, 6%, all mild). 44 (92%) experienced eyestrain (17 frequently, 27 occasionally).	5
Lindberg et al.	2020	Denmark	188 Danish eSports athletes	184 males, 4 females	Mean: 17.1 ± 2.3	Prevalence of MSK pain, impact of training volume and physical activity level on pain	80 athletes (42.6%) reported pain in the past week; most sites: back (31.3%), neck (11.3%), shoulders (11.3%). 6.25% had pain that limited eSports participation. 32% pain at one site, 27% at two sites and 9% at three sites. Median number of pain sites: 2 (range 1–13), mean pain intensity: 4 (SD: 1.8). Athletes with pain significantly lower weekly eSports training volume (20.9 ± 15.1 h) compared to those without pain (26 ± 18.5 h) (mean difference: 5.6 h, *p* = 0.027). No significant difference in weekly physical activity levels between those with pain (3722 ± 3667.3 MET‐minutes) and those without (3641.3 ± 4563.1 MET‐minutes) (mean difference: 81.1 MET‐minutes, *p* = 0.906).	7
Basuodan et al.	2023	Saudi Arabia	95 eSports athletes who took part in the Saudi eSports Federation	80 males, 15 females	Mean: 24.6 ± 4.2	Explore carpal tunnel syndrome, hand symptoms and their severity	14 (15%) reported carpal tunnel syndrome; control group (non‐eSports athletes) had 4 (4%). Males had significantly higher scores for hand (*p* < 0.1) and radiating symptoms (*p* < 0.1) compared to females. Athletes with >10 years of experience had significantly higher BCTQ‐SSS scores compared to those with <10 years (*p* = 0.003). Earlier career starts correlated with higher BCTQ‐SSS scores (*p* = 0.004). Radiating hand symptoms were found only in athletes who did not use armrests (27.27%). No athlete using armrests reported radiating hand symptoms.	7
Fathuldeen et al.	2023	Saudi Arabia	116 eSports athletes in Saudi Arabia	100 males, 16 females	Mean: 22.5 (range: 18−48)	Prevalence of musculoskeletal injuries, awareness of competitive video gamers about these injuries	27 (23.3%) stopped gaming due to injury, 8 (6.9%) required medical consultation for an injury, 6 (5.2%) were diagnosed with a medical condition related to gaming. 24 (20.7%) suffer from a bowed back. Musculoskeletal injuries (MSIs) were reported by all female participants versus 84% of males (*p* = 0.048). MSIs were more common among those gaming 1–5 years (100%) compared to those with >10 years (80.8%) (*p* = 0.045). Those playing <10 days/month had more MSIs than those playing 20–30 days (*p* = 0.049). 100 (86.2%) participants with at least one MSI; 16 (13.8%) had none. Most sites: lower back (63.8%), neck (50%), hand/wrist (44.8%), shoulder (35.3%), upper back (27.6%), elbow (12.9%). 50.4% believed gaming tournaments negatively affect the musculoskeletal system; 43 (37.1%) linked tournaments to conditions like tendinopathy, carpal tunnel syndrome and repetitive stress injuries.	4
DiFrancisco‐Donoghue et al.	2019	USA	65 collegiate varsity eSports athletes	Unknown	Between 18 and 22 years	Health issues in competitive gaming	The most complaint: eye fatigue (52%), back and neck pain (41%), wrist pain (36%) and hand pain (30%). Injuries in eSports are similar to those seen in sedentary office workers. 2% of athletes who experienced an ailment sought medical attention.	3
Kurniawan et al.	2024	Indonesia	43 eSports athletes	42 male, 1 female	36 < 25 years, 7 > 25 years	Prevalence of musculoskeletal problems among professional gamers	The majority of professional gamers players experienced musculoskeletal complaints (65.1%, *n* = 28). Injuries in the shoulder (13), hand (12), neck (10), wrist (7) and elbow/lower back (2 each). Eye fatigue in 19 cases.	6
Ekefjärd et al.	2024	Sweden	40 eSports athletes	Unknown	18−22 = 47.5%, 23−27 = 32.5%, 28 or older = 20	Prevalence of self‐reported physical symptoms	25 (62.5%) at least one physical symptom related to gaming 3 months before the survey. Most complaints: headache (40%), eye‐related issues (32.5%, e.g., fatigue or dryness), wrist pain (12.5%), lower back pain (7.5%), shoulder pain (7.5%), elbow pain (5%), hand pain (5%) and finger pain (2.5%). A significant association between playing time and physical symptoms (OR = 8.0; 95% CI 1.4–44.6, *p* = 0.018), with more than 35 h of weekly gaming linked to eight times higher odds.	5
Monma et al.	2024	Japan	39 eSports athletes	All male	Mean: 21.6 ± 5.6	Prevalence of physical complaints and their association with eSports activities among Japanese eSports players.	Most physical complaints in professional gamers: 25 (64.1%) eye fatigue, 23 (59%) stiff or sore shoulders, 16 (41%) neck pain, 16 (41%) lower back pain, 12 (30.8%) headache, 5 (12.8%) wrist pain and 2 (5.1%) finger pain. Professional gamers significantly more likely to experience neck pain (*p* < 0.05), wrist pain (*p* < 0.05) and lower back pain (*p* < 0.05) compared to amateurs.	6
Lam et al.	2022	China	48 mobile eSports athletes from Onmyoji Arena Pro League	All male	Mean: 20.1 (SD: 1.67, range: 18–24)	Posture, mobility and stability of the spine among top eSports athletes	Mobile eSports athletes had significantly worse spinal posture, mobility and stability.	6

Abbreviations: BCTQ‐SSS, Boston Carpal Tunnel Questionnaire Symptom Severity Scale; CI, confidence interval; MET, metabolic equivalent of task; OR, odds ratio; SD, standard deviation.

### Information regarding complaint prevalences

Table 2 includes the complaint region, pain severity, failures and healthcare aspects along with prevalences or total numbers.

**Table 2:** Complaint prevalences, pain intensity, functional impairments and healthcare‐related measures.

Different aspects	Prevalence (%)	Total (*n*)
Anatomical region		
General prevalence	26.8% [4]−86.2% [8]	
Head	30.8% [22]−40% [7]	
Neck	11.3% [19]−50% [8]	
Upper back	24% [18]−27.6% [8]	
Lower back	4.7% [16]−63.8% [8]	
Wrist	8% [18]−44.8% [8]	
Hand	5% [7]−30% [6]	
Fingers	2.5% [7]−38% [18]	
Thumb	5.3% [4]	
Carpal tunnel	15% [1]	
Shoulder	2.7% [4]−59% [22]	
Knee	6% [18]	
Eye fatigue	32.5% [7]−92% [18]	
Elbow	4.7% [16]−12.9% [8]	
Pain severity		
Mean pain intensity scale^a^		4 (SD: 1.8) [19]
General pain intensity^b^		82 mild, 10 moderate [18]
Failures		
Time loss from eSports	4.6% [4]−23.3% [8]	
Average of missed competition time		3.0 ± 2.3 weeks (range = 1–8 weeks) [4]
Healthcare aspects		
Physician contacts	2% [6]−6.9% [8]	

*Note*: In cases where multiple studies have reported a prevalence in relation to an anatomical region, the range of the smallest and largest reported values will be provided and therefore two citations are given there. Conversely, if only a single study has reported a prevalence, only the value from that study is cited in isolation. Abbreviation: SD, standard deviation.

^a^ Numeric pain rating scale: 0–10.

^b^ Graded by a 10‐point Likert scale: nil 0, mild 1–3, moderate 4–6, severe 7–9.

### Physical complaints by body region

The reported data presents the prevalence of complaints initiated as a consequence of the professional practice of eSports as reported in diverse body regions across a range of studies (see Table 2). The most frequently reported anatomical regions affected by musculoskeletal complaints were the shoulder [4, 19, 20, 21, 22], neck [4, 6, 8, 19] and wrists [22, 23, 24, 25, 26, 27, 28]. Furthermore, with marginally lower frequencies a number of studies have documented complaints in the lower back [7, 22] and elbows [4, 7, 16]. Beyond musculoskeletal symptoms, eye fatigue was also commonly mentioned [16, 22] [6, 7].

### Spinal health

Lam et al. [17] showed in a retrospective case series that mobile eSports athletes had substantially worse spinal posture (*p* < 0.003), mobility (*p* < 0.001) and stability (*p* < 0.001). However, no correlations were found between spinal assessment outcomes and factors including body mass, height, BMI, career duration or team membership [17].

### Injuries, severity and medical contacts

Clements et al. [4] found that a total of 41 e‐athletes (26.8%) experienced at least one injury, with 17 e‐athletes (11.1%) reporting multiple injuries initiated by practicing eSports professionally. Longer team membership (*p* = 0.03) and practicing more than 5 h per day (31.7% injured vs. 10.7% uninjured, *p* = 0.01) had a higher injury prevalence with the wrist, neck and back as the most frequently affected region. No significant differences were found in age, sex, scholarship status, age of starting eSports or days per week playing eSports [4].

In terms of severity, the average rating on the numeric pain rating scale (NPRS) in a study by Lindberg et al. [19] was 4 (0 = no pain; 10 = worst possible pain). Lam et al. [18] classified the intensity of pain into four categories (graded by a 10‐point Likert scale: nil 0, mild 1–3, moderate 4–6, severe 7–9), ranging from mild to severe. Of the participants, 82 reported experiencing mild pain, while 10 stated that their pain was moderate in intensity [18].

For the treatment and examination of complaints related to eSports, a medical consultation was reported by 2% [6] to 6.9% [8] of the eSports players. In total 5.2% of participants have ever been diagnosed with a medical condition related to playing video games [8]. Furthermore, between 4.6% [4] and 23.3% [8] of eSports players stated that their eSports career had already been interrupted due to injury. The duration of competition time missed due to injury was reported by Clements et al. [4] to be between 2 and 8 weeks and was caused in particular by disorders to the wrist, thumb and elbow.

### Different aspects of musculoskeletal complaints

In a survey by Fathuldeen et al. [8], 50.4% of participants stated that they believed there were negative effects on the musculoskeletal system from playing electronic games. Furthermore, Kurniawan et al. [16] observed that 65.1% of professional gamers experienced musculoskeletal complaints. Monma et al. [22] found that professional eSports players were substantially more likely to experience neck pain (*p* < 0.05), wrist pain (*p* < 0.05) and lower back pain (*p* < 0.05) compared to amateur players.

A connection between eSports competitions and complaints, including tendinopathies, carpal tunnel syndrome or repetitive strain injuries was suspected by 37.1% of the eSports players surveyed [8]. DiFrancisco‐Donoghue et al. [6] found that the injuries observed in eSports athletes, including those affecting the hands, wrists, eyes and neck, are similar to those seen in sedentary office workers [6].

A recent survey Lindberg et al. [19] showed a significant correlation between musculoskeletal pain and reduced eSports‐related training volume. The weekly training volume was 5.6 h lower in the group with musculoskeletal pain during the previous week (20.9 ± 15.1 h) compared to the group without musculoskeletal pain (26 ± 18.5 h). This difference was statistically significant (95% CI: 10.6–0.7, *p* = 0.027). However, no significant difference was observed in the average weekly physical activity levels between the two groups. The mean metabolic equivalent of task (MET) minutes per week score from the international physical activity questionnaire short form for those reporting MSK pain was 3722 ± 3667.3, compared to 3641.3 ± 4563.1 for those with no pain, indicating a mean difference of 81.1 MET‐minutes per week (95% CI: −1266.9 to 1429.1, *p* = 0.906) [19].

In terms of career duration, all participants who had been playing video games for a period of between 1 and 5 years reported musculoskeletal disorders, while this applied to 80.8% of those who had been playing for more than 10 years (*p* = 0.045). Those who engaged in video gaming for less than 10 days per month exhibited a prevalence of musculoskeletal disorders, while 83.1% of those who engaged in gaming for 20–30 days per month also showed this outcome (*p* = 0.049) [8].

Furthermore, e‐athletes with over 10 years of experience exhibited elevated Boston Carpal Tunnel Questionnaire Symptom Severity Scale (BCTQ‐SSS) scores (*p* = 0.003), and an earlier career commencement was associated with higher scores (*p* = 0.004). The presence of radiating hand symptoms was found exclusively in athletes who did not use armrests (27.27%), with no symptoms reported in those who did employ armrests [1].

A comparable result was observed in another study [7], which showed a notable correlation between playing time and the occurrence of physical symptoms (OR = 8.0; 95% CI: 1.4–44.6, *p* = 0.018). In particular, playing for more than 35 h per week was found to be substantially associated with an eightfold increase in the likelihood of experiencing physical symptoms [7].

### Quality assessment

NOS adapted for cross‐sectional studies and adapted for case series were used. For the cross‐sectional studies, the quality score ranged from 3 to 7. In summary, two studies did not achieve satisfactory quality, five studies showed satisfactory quality and two studies showed good quality in the quality assessment.

The single retrospective case‐series was assessed with six points based on the adapted NOS criteria and achieved a MQ.

The results of the risk of bias assessment are presented in Table 1. A detailed overview is shown in Table 3.

**Table 3:** Newcastle–Ottawa scale adapted for cross‐sectional studies (surveys) and for case series.

Cross‐sectional study (survey)—NOS adapted for cross‐sectional studies (surveys) [12]										
	Selection				Comparability		Outcome			Score
Publication	Representative of exposed cohort	Sample size justified	Nonrespondents comparability	Ascertainment of exposure	Control for important factors	Additional factors	Outcome assessment	Appropriate statistical tests		
Clements et al. [4]	Yes	Yes	No	Yes	No	Yes	Yes	Yes		6
Lam et al. [17, 18]	Yes	No	No	Yes	No	Yes	Yes	Yes		5
Lindberg et al. 2020	Yes	Yes	No	Yes**	No	Yes	Yes	Yes		7
Basuodan et al. [1]	Yes	Yes	No	Yes	No	Yes	Yes**	Yes		7
Fathuldeen et al. [8]	Yes	Yes	No	Yes	No	No	Yes	No		4
DiFrancisco‐Donoghue et al. [6]	Yes	No	No	Yes	No	No	Yes	No		3
Kurniawan et al. [16]	Yes	Yes	No	Yes	No	Yes	Yes	Yes		6
Ekefjärd et al. [7]	Yes	No	No	Yes	Yes	No	Yes	Yes		5
Monma et al. [22]	Yes	Yes	No	Yes	Yes	No	Yes	Yes		6

Retrospective case‐series—NOS adapted for case series [23]										
	Representative of exposed cohort	Ascertainment of exposure	Ascertainment of outcome	Were alternative causes ruled out	Control for important factors	Additional factors	Outcome assessment	Follow‐up	Sufficient case details	
Lam et al. [17, 18]	Yes	No	No	Yes	Yes	Yes	Yes	No	Yes	6

*Note*: In the categories marked in red it is possible to achieve two points. This was achieved in the studies marked with an **. Abbreviation: NOS, Newcastle‐Ottawa Scale.

## DISCUSSION

The most important finding of this systematic review is that musculoskeletal disorders are a common problem in eSports. The wide range of reported prevalence rates likely reflects methodological differences between studies rather than true epidemiological variation alone and suggests that the overall burden may be underestimated. Nevertheless, the anatomical region–specific prevalence ranges presented in Table 2 provide a transparent representation of this variability without implying artificial homogeneity. While the general prevalence of musculoskeletal disorders in eSports players is up to 86.2% [8], the lower back, neck and shoulders are particularly affected [8, 22]. Although not being classified as a musculoskeletal disorder eye fatigue was also analysed as a very frequent functional problem [18]. These disorders not only result in a significant loss of training time [19], but also an important amount of eSports athletes experienced a loss of competition time [4]. Musculoskeletal disorders are much more prevalent in eSports athletes than in the general population [37], which is particularly worrying as only very young players were included in the studies. The majority of them were male and the average age of this specific target group was of 17–24 years. As no upper age limit was specified for the selection of athletes in the studies, it can be hypothesised that the high cognitive demands of eSports facilitate a professional career in eSports primarily at a younger age.

Despite the numerous benefits of eSports, including the promotion of cognitive skills and social interaction [10, 28], it is associated with specific health risks [15]. Extended periods of sitting and high‐intensity repetitive movements, particularly of the upper limb, expose eSports players to musculoskeletal disorders [33]. An ergonomically unfavourable gaming setup without armrests results in poor posture [1, 2, 3, 4, 5, 6, 7, 8, 9, 10, 11, 12, 13, 14, 15, 16, 17]. These factors lead to increased strain on the musculoskeletal system. A further challenge is the education of eSports players regarding the development of musculoskeletal disorders. For example, only 37.1% of the surveyed eSports athletes believed that there was a connection between the occurrence of tendinopathies, carpal tunnel syndromes and injuries caused by repetitive strain from playing eSports [8].

The data regarding career duration and gaming hours regarding the onset of musculoskeletal complaints are heterogeneous. Ekefjärd et al. reported a significant positive correlation between the number of hours spent gaming and the occurrence of musculoskeletal complaints. In particular, a weekly gaming workload exceeding 35 h was associated with an eightfold increased risk of developing musculoskeletal complaints [7]. Basuodan et al. also identified an elevated risk of upper‐extremity neuropathies in eSports athletes as gaming hours increased, though this finding was not statistically significant. In contrast, the frequency of upper extremity neuropathies significantly increased with longer career durations [1].

Fathuldeen et al., however, reached opposing conclusions. In their study, both the duration of gaming time and the number of monthly gaming days were inversely correlated with the occurrence of musculoskeletal complaints. A career duration of 1–5 years was associated with a higher prevalence of musculoskeletal complaints compared to a career lasting over 10 years. One potential explanation for this could be adaptive processes, including individual compensation strategies. Another assumption is that eSports athletes who experience fewer or less severe complaints may be more likely to have longer careers [8]. However, it is important to note that the methodological limitations of the underlying study (e.g., lack of a control group, high margin of error, small sample size, especially among eSports athletes with a career duration of 1–5 years, and limited access to the population) may restrict the generalisability and interpretability of these results.

Beyond the musculoskeletal system internal diseases and mental health challenges also play a significant role in the professional eSports. In a survey conducted by Monteiro Pereira et al. in 2021 [27], 37% of eSports athletes presented symptoms of anxiety and depression, while 45% reported experiencing sleep disorders. Furthermore, various of effects on the cardiovascular system are discussed regarding the practice of professional eSports. Increased activity of the sympathetic nervous system is suspected as a result of competitive games [35], and the significant increased expression of subjective stress and reduced heart rate variability after defeats indicate a potentially increased cardiovascular stress in professional e‐athletes [20].

As a result, eSports athletes will increasingly represent a proportion of patients in sports medicine clinics and practices, it is essential for healthcare professionals to be aware of the associated risks and take appropriate measures to address these concerns. Sports physicians must be conscious of the specific health risks associated with e‐athletes and integrate these considerations in their medical histories and examinations. In addition to providing comprehensive information on specific health risks, it is essential for sports physicians to give advice on the development and implementation of individualised prevention strategies.

With regard to preventive measures, it is recommended that eSports are practiced at an ergonomically designed gaming workstation. The correct adjustment of the chair, table, screen and in particular the use of armrests, can effectively prevent musculoskeletal disorders [1]. Before starting an eSports session, it is recommended to establish a warm‐up routine and regular breaks [34]. Furthermore, it is recommended for eSports players to engage in complementary physical activities that improve endurance and muscle strength alongside eSports as a form of compensatory training [24].

This Review supports our hypothesis that musculoskeletal disorders are prevalent among professional eSports athletes and confirms the urgent need for preventive measures. To facilitate the early diagnosis of eSports‐related health concerns, regular medical check‐ups with a comprehensive approach considering physical, psychological and ergonomic assessments for eSports athletes are strongly recommended.

## LIMITATIONS

Several limitations of the review should be noted. A total of ten studies with a total number of 789 analysed eSports players were identified. This shows that there are only a few studies with small study populations that provide information on musculoskeletal disorders in professional eSport players in different countries. The definition of the term ‘professional’ e‐sports player varied across studies (e.g., collegiate players, elite mobile gamers, professional league athletes). This heterogeneity likely contributed to the variability in reported prevalence rates and limits the generalisability of the findings. Even when descriptively considering sample sizes, prevalence estimates remain highly variable, suggesting that methodological differences rather than sample size alone account for the wide range. It must be noted that the eSports players who were examined were predominantly male and very young. The predominance of young male participants limits the transferability of findings to female or older eSports athletes. Importantly, most included studies relied on self‐reported symptoms rather than clinically confirmed diagnoses. Therefore, the reported prevalence reflects symptom burden rather than medically verified musculoskeletal disorders, which may result in over‐ or under‐estimation. Furthermore, the included studies were almost exclusively cross‐sectional studies, meaning that causal relationships and potential confounders such as prior injuries, ergonomic setup, physical activity level or psychosocial stressors could not be established. In addition, a large proportion of the eSports participants were from Asia. The results of the review, therefore, do not represent the entire eSports community. A further limitation of the review is that none of the included studies conducted a follow‐up, and it was not possible to categorise musculoskeletal disorders as acute or chronic due to a lack of data.

## CONCLUSIONS

Musculoskeletal disorders are common among professional eSports athletes, impacting performance and causing inactivity, yet sports medicine treatment remains a rarity. In the daily clinical practice, structured screening for neck, shoulder and wrist complaints should be considered in this population. Preventive strategies should combine ergonomic optimisation, targeted strengthening programs and interdisciplinary collaboration between sports physicians, physiotherapists and ergonomics specialists. Further strategies like warm‐up routines, regular game breaks with physical activity and ergonomic setups are key. High‐quality research is needed for evidence‐based prevention and causal relationships between musculoskeletal disorders and the practice of eSports.

## AUTHOR CONTRIBUTIONS

*Study conception and design*: Ludwig Schlesiger, Jakub Oronowicz, Christoph Lutter, Thomas Tischer, Romain Seil and Andrzej Jasina. *Data collection and extraction*: Ludwig Schlesiger, Jakub Oronowicz and Andrzej Jasina. *Data analysis and interpretation*: Ludwig Schlesiger, Jakub Oronowicz, Andrzej Jasina, Thomas Tischer and Christoph Lutter. *Drafting of the manuscript*: Ludwig Schlesiger, Jakub Oronowicz and Christoph Lutter. *Critical revision of the manuscript*: Thomas Tischer, Romain Seil and Christoph Lutter. All authors approved the final version of the manuscript.

## CONFLICT OF INTEREST STATEMENT

The authors declare no conflicts of interest.

## ETHICS STATEMENT

This study did not involve human participants. Ethical approval was therefore not required. Patients and/or the public were not involved in the design, conduct, reporting or dissemination plans of this research. This systematic review was registered in the PROSPERO database (CRD42024599915) and conducted according to the Preferred Reporting Items for Systematic Reviews and Meta‐Analyses (PRISMA 2020) checklist.

## Data Availability

No additional data are available.
